# Supplementary material for: The interactome of intact mitochondria by cross-linking mass spectrometry provides evidence for coexisting respiratory supercomplexes
Source: Mol Cell Proteomics. 2017 Dec 8;17(2):216–32. doi: 10.1074/mcp.RA117.000470 (PMC5795388; doi:10.1074/mcp.RA117.000470)
Supplement: Supplemental Data [file supp_RA117.000470_133922_0_supp_23978_fzffwf.zip › spectra_annotation/mito_DR_spectra_annotation/84-1-3-1-19-1.pdf]

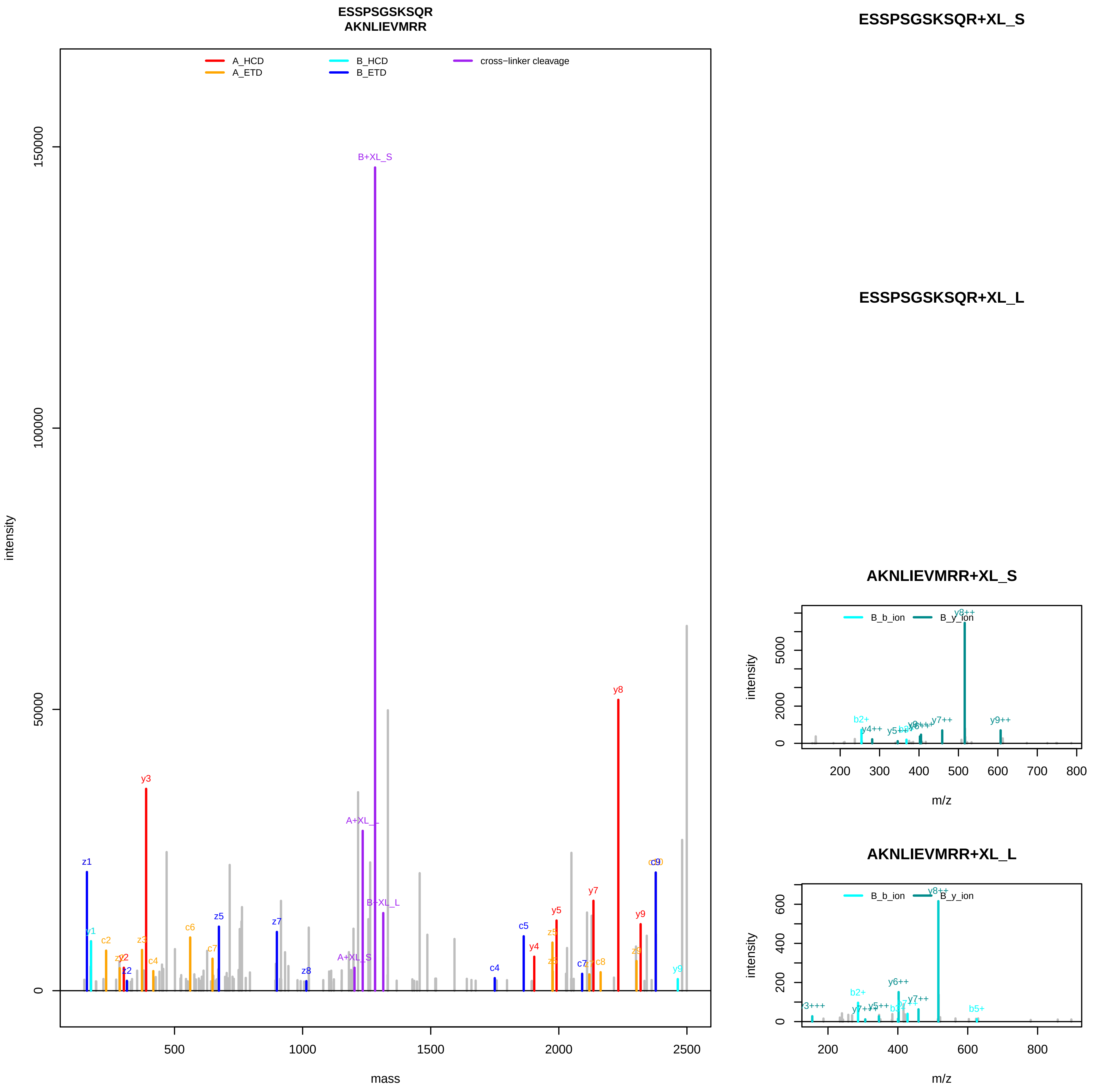
ESSPSGSKSQR
AKNLIEVMRR
A_HCD
A_ETD
B_HCD
B_ETD
cross−linker cleavage
intensity
mass
0
50000
100000
150000
500
1000
1500
2000
2500
B+XL_S
A+XL_L
B+XL_L
A+XL_S
y3
y8
y9
y7
y5
y4
z1
y1
c2
c4
c6
c7
z5
z7
z8
c4
c5
c7
c8
c9
y9
ESSPSGSKSQR+XL_S
ESSPSGSKSQR+XL_L
AKNLIEVMRR+XL_S
B_b_ion
B_y_ion
intensity
m/z
0
2000
5000
200
300
400
500
600
700
800
y8++
b2+
y4++
y7++
y9++
AKNLIEVMRR+XL_L
B_b_ion
B_y_ion
intensity
m/z
0
200
400
600
800
y8++
b2+
y6++
y7++
b5+
